# Sorption and Diffusion of Water Vapor and Carbon Dioxide in Sulfonated Polyaniline as Chemical Sensing Materials

**DOI:** 10.3390/s16050606

**Published:** 2016-04-27

**Authors:** Qiuhua Liang, Junke Jiang, Huaiyu Ye, Ning Yang, Miao Cai, Jing Xiao, Xianping Chen

**Affiliations:** 1 Faculty of Electromechanical Engineering, Guilin University of Electronic Technology, Guilin 541004, China; lqh91@outlook.com (Q.L.); jiangjunke92@outlook.com (J.J.); joeyoung0014@163.com (N.Y.); caimiao105@163.com (M.C.); xiaojing@guet.edu.cn (J.X.); 2 Key Laboratory of Optoelectronic Technology & Systems, Education Ministry of China, Chongqing University and College of Opto-electronic Engineering, Chongqing University, Chongqing 400044, China; huaiyuye@163.com

**Keywords:** polyaniline, sensitivity, adsorption, diffusion, gas sensors

## Abstract

A hybrid quantum mechanics (QM)/molecular dynamics (MD) simulation is performed to investigate the effect of an ionizable group (–SO_3_^−^Na^+^) on polyaniline as gas sensing materials. Polymers considered for this work include emeraldine base of polyaniline (EB-PANI) and its derivatives (Na-SPANI (I), (II) and (III)) whose rings are partly monosubstituted by –SO_3_^−^Na^+^. The hybrid simulation results show that the adsorption energy, Mulliken charge and band gap of analytes (CO_2_ and H_2_O) in polyaniline are relatively sensitive to the position and the amounts of –SO_3_^−^Na^+^, and these parameters would affect the sensitivity of Na-SPANI/EB-PANI towards CO_2_. The sensitivity of Na-SPANI (III)/EB-PANI towards CO_2_ can be greatly improved by two orders of magnitude, which is in agreement with the experimental study. In addition, we also demonstrate that introducing –SO_3_^−^Na^+^ groups at the rings can notably affect the gas transport properties of polyaniline. Comparative studies indicate that the effect of ionizable group on polyaniline as gas sensing materials for the polar gas molecule (H_2_O) is more significant than that for the nonpolar gas molecule (CO_2_). These findings contribute in the functionalization-induced variations of the material properties of polyaniline for CO_2_ sensing and the design of new polyaniline with desired sensing properties.

## 1. Introduction

Polyaniline is one of the most intensively studied conducting polymers due to its ease of synthesis, tunable properties, reversible insulator-to-metal transitions, electrochromic behavior and good environmental stability [1,2,3]. These unique features make polyaniline widely applicable in different areas, including transistors [4], optical devices [5], light emitting diodes [6], supercapacitors [7], rechargeable batteries [8], corrosion protection [9], electromagnetic interference shielding [10], electrochromic displays [11], gas separation [12], water harvesting [13], *etc*. However, the insolubility of polyaniline in aqueous solution and most common organic solvents hinders its applications [14]. With the environmental concerns, the polymer materials should be able to be processed in a water medium without using any other solvents [3,14,15]. Synthesis of polyaniline in basic solutions is an economical and environmentally-friendly solution [16]. The good solubility also ensures polyaniline for easy post-processing, for example, by inexpensive fiber spinning or spin coating [17]. Introducing ionizable groups, such as carboxylic acid [18], sulfonic acid [14,15,17,18,19], and boronate groups [20], onto the backbone is the most successful approach to increase the solubility of polyaniline in aqueous solution without sacrificing its conductivity [12]. These ionizable groups can dissociate into basic aqueous to make polyaniline have polyelectrolyte properties [19]. The hydrophilic interactions between the covalently attached ionized groups and polar molecules of water overcome the interchain interactions along the polyaniline chains, which allow the rapid solvation of the polyaniline backbone [3]. The development of basic polyaniline as gas sensors for monitoring the environmental pollutants, such as HCl [21], H_2_S [22], CO_2_ [23], CO [24], NH_3_ [25], NO_2_ [26], H_2_ [27,28,29], methanol [30,31], and chloroform [32] has been extensively conducted. Theoretical works [2,18] and experimental studies [33] have demonstrated that the solubility of polyaniline in water is significantly improved by introducing –SO_3_^−^Na^+^ or –SO_3_^−^K^+^ group on the backbone of polyaniline. In addition, distinctive properties, such as electroactivity and conductivity over a wider pH range, and improved sensitivity have been discovered with these sulfonated polyaniline.

Substantially theoretical investigations [2,18,34,35] have been conducted to understand the sensing behavior of polyaniline. Kaner *et al.* [27,28] have investigated the polyaniline sensing performance towards H_2_ gas, in which the interaction between hydrogen of H_2_ and nitrogen atoms on two adjacent chains may form a bridge or there may be protonation the nitrogens in imine group partially. Ostwal *et al.* [12,13] have demonstrated that doping polyaniline with different acid (HCl and HBr) can significantly affect the self-diffusivity of water vapor in the polymer. With quantum mechanics (QM) calculations, Ullah *et al.* [35] have investigated the interactions of emeraldine salt (ES) with both O (CO(1)) and C (CO(2)) sides of CO to observe the differences such as interaction energy, counterpoise-corrected energy, and Mulliken charge analysis. Chen *et al.* [2,18] studied the effect of the number of ionizable groups on the interaction energies of the analytes with the polyaniline by Grand Canonical Monte Carlo (GCMC) and molecular dynamics (MD). The GCMC and MD approaches are fast, which are good for candidate screening with big-size systems model. Our previous study [36] has shown that material properties of carbon nanotubes (CNT) are affected by the distribution of functional groups on the CNT surface. For gas sensing of polyaniline, it is expected that the position of ionizable groups on polyaniline backbone may affect the interactions between analyte molecules and polymers. More accurate calculation about the effect of the position of the ionizable groups on the polymer-analyte interactions by QM is thus necessary. These information will also help in studying the transport of small molecules, such as H_2_O and CO_2_, in the polyaniline matrix.

**Figure 1:**
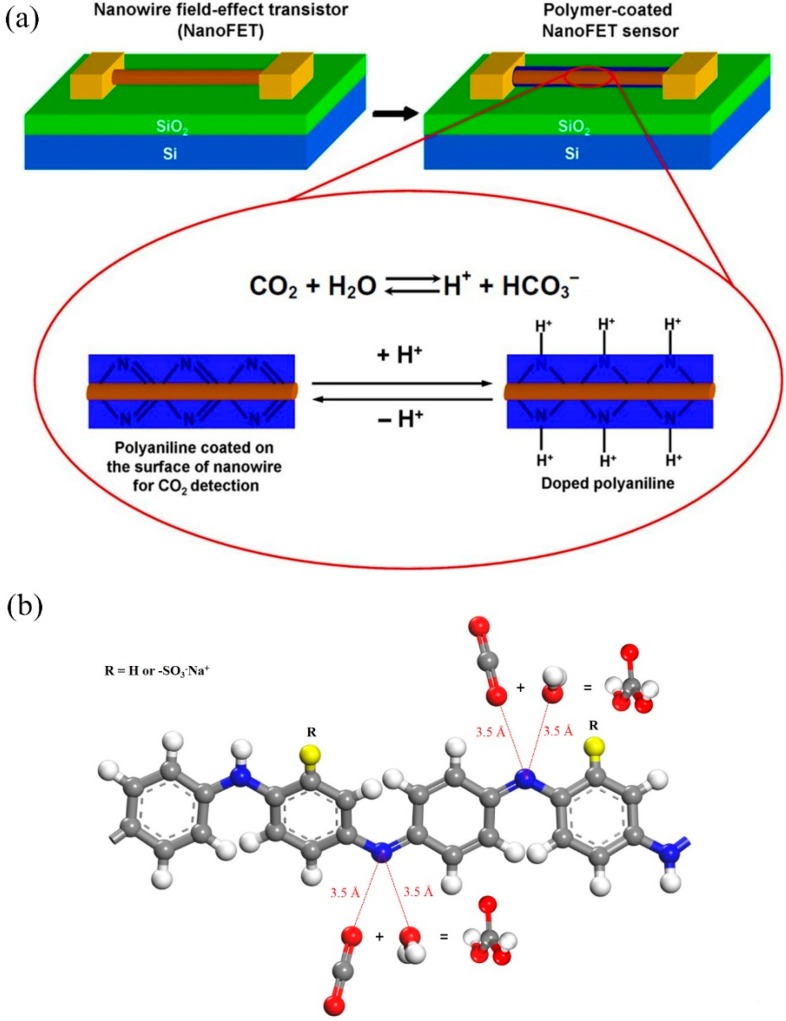
(**a**) schematic diagram of sensing mechanism of an example polyaniline (PANI) coated nanowire field-effect transistor (NanoFET) as CO_2_ sensor; (**b**) examples of CO_2_ react with H_2_O adsorbing in the sites of PANI to form H_2_CO_3_.

Based on the different types of the gas, the response mechanism can consist of protonation, deprotonation, reduction, *etc.* [37]. The sensing mechanism of the polyaniline as CO_2_ sensor mainly proceeds in two steps: firstly, the CO_2_ reacts with H_2_O to form carbonic acid (H_2_CO_3_) which dissociates into H^+^ and HCO_3_^−^; then, protonic acid doping of the insulated polyaniline forms the conductive counterpart. As shown in Figure 1a, the polyaniline is coated on the nanowire field-effect transistor (NanoFET) as sensing element. When the polyaniline detects the analyte molecules, the resistance of polyaniline (PANI) would change and the NanoFET can detect and transfer the resistance change to other detectable physical signal-currents [18]. The differences of CO_2_ concentrations directly impact the doping degree, thus resulting in the differences of the resistance of PANI-coated nanowire, and, thereby, the concentration of CO_2_ can be measured. As shown in Figure 1b, when the analytes diffuse and are adsorbed in the PANI system, the more analytes adsorb in the site of imine, and the more H_2_CO_3_ will be created along with the chemical reaction. In addition, the sensitivity of polyaniline towards H_2_CO_3_ is also an important performance as a sensing material. Experimental works [23,38] exhibit a trend of improving the sensitivity of the polymer towards analytes by modifying the molecular structure and the chemical composition.

Different from the previous paper, which investigated the effect of functional groups on the working range of polyaniline as CO_2_ sensors by MD, the objective of this article is to study the effect of charge transfer, adsorption site and band gap on the sensing performance of emeraldine base of polyaniline (EB-PANI) and its derivatives (Na-SPANI I, II and III) whose rings are monosubstituted by ionizable groups –SO_3_^−^Na^+^. Adsorption and diffusion of H_2_O and CO_2_ in EB-PANI, Na-SPANI (I), Na-SPANI (II) and Na-SPANI (III) are also investigated. Understanding these issues will help us to know which adsorption sites and which adsorbents are more capable of adsorbing more CO_2_ and H_2_O, and thereby forming more H_2_CO_3_. In addition, the increasing sensitivity of Na-SPANI/EB-PANI towards H_2_CO_3_ is also evaluated. In the present study, all of the calculations are employed in periodic 3D atomistic models of the polymers. A detailed density functional theory (DFT) study of the interaction energy and Mulliken charge of H_2_O and CO_2_ adsorbed in polymers has been conducted. A new equilibrating protocol derived from our previous study [39] is utilized to create 3D atomistic models with realistic density and low-potential energy characteristics of the polymer. The sorption isotherms of H_2_O and CO_2_ for each of the structures have been computed to understand the adsorbing number changes of analytes for the influence of site and number of functional group. The diffusion coefficients of H_2_O and CO_2_ in EB-PANI and Na-SPANI (III) are calculated to know the diffusion situation of analytes in the polyaniline system.

## 2. Materials and Methods

All the simulations were performed using the commercial software Materials Studio^®^ 7.0 (BIOVIA, San Diego, CA, USA). The Forcite Plus module was used for molecular mechanics and MD simulation, while, for QM calculation, the DMol^3^ module was implemented. According to our previous study on validation of forcefields in predicting the physical and thermophysical properties of emeraldine base polyaniline [39], it was encouraging to see that the second generation forcefield Condensed-phase Optimized Molecular Potentials for Atomistic Simulation Studies (COMPASS) can provide more accurate precision in determining the polyaniline properties under experimental conditions than estimation by Polymer Consistent Forcefield (PCFF). Therefore, COMPASS was employed for all the molecular mechanics (MM) and MD simulations in this study.

### 2.1. Atomistic Models

**Figure 2:**
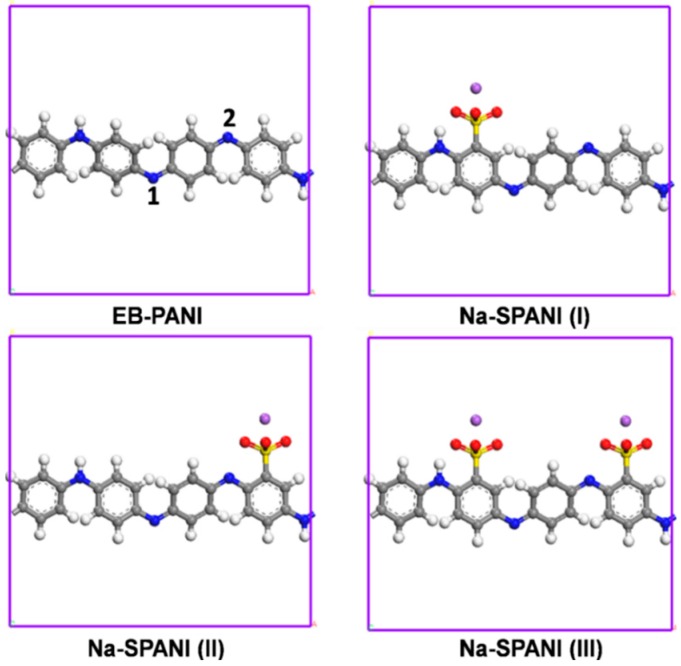
The monomers and adsorption sites of EB-PANI, Na-SPANI (I), Na-SPANI (II) and Na-SPANI (III) in periodic structure.

**Figure 3:**
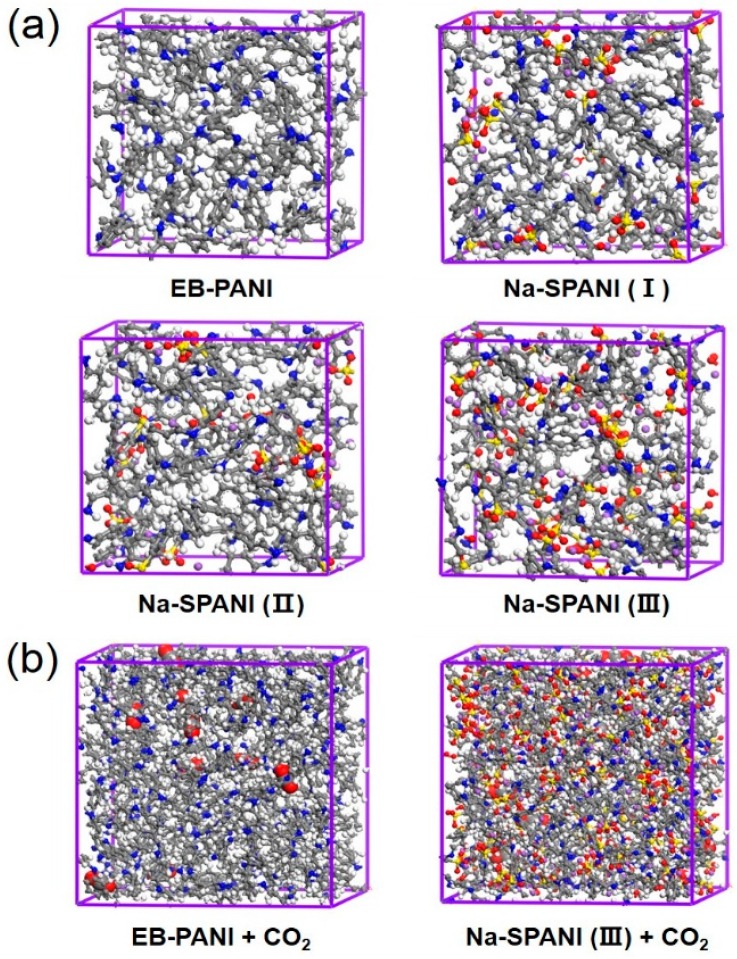
(**a**) the atomistic modeling of EB-PANI, Na-SPANI (I), Na-SPANI (II) and Na-SPANI (III); (**b**) CO_2_ diffusion in the EB-PANI and Na-SPANI (III).

Four types of polyanilines were examined as the sensor candidates in this study. For each of the polymers, two adsorption sites were considered (Figure 2). The polymer molecules were generated by using the polymer builder based on its stereoisomerism (tacticity) and sequence isomerism (connectivity). The tacticity of the repeat units constructed by the polymer builder was isotactic. The side groups could be irregularly distributed on one side (isotactic), alternate sides (syndiotactic) of the polyaniline. The torsion angle between the repeat units will be generated at random range from −180 to 180. The connectivity of the monomer units was head-to-tail [40]. Figure 3a illustrates the periodic molecular models for adsorption isotherm calculations. Two polymers, the EB-PANI and Na-SPANI (III), were employed in the diffusion study. The periodic models for the CO_2_ diffusion in EB-PANI and Na-SPANI (III) calculations were given in Figure 3b. The polymer models of EB-PANI, Na-SPANI (I), Na-SPANI (II) and Na-SPANI (III) for sorption contained 928, 1008, 1008 and 1088 atoms, respectively, and the corresponding cubic boxes of were 21.5 Å, 22.5 Å, 22.5 Å and 23.30 Å, respectively. For the calculation of CO_2_ diffusion in EB-PANI and Na-SPANI (III), the atomistic atoms were 3726 and 4366, and the cubic boxes were 34 Å and 37 Å, respectively.

The effect of the ionizable groups and its positions on adsorption energy, charge transfer, band gap and sensitivity of the analytes to sensing material were studied. The adsorption energy refers to the physical interaction between the analyte-polyaniline. All of the adsorption energy, charge transfer and band gap directly contribute to the sensitivity of the sensor. The macroscopic molecular mechanics model has been adopted in the adsorption energy calculation. Charge transfer between analytes and polyaniline could result in the change of electronic properties and the band gap of polyaniline, which can reflect the sensitivity of interaction [41]. In order to evaluate the response speed of the candidates, diffusion coefficients of CO_2_ in the EB-PANI and Na-SPANI (III) polymer matrixes have been calculated.

### 2.2. Simulation

Sensitivity, which indicates the ability of sensing material to input change, was the key concern in gas sensing. Resolution and response speed were the most important aspects for molecular design of sensor candidates [42]. Adsorption of analytes on sensing materials tackles the resolution concerns while the diffusion of analyte along the sensing material provides us the response time of the sensor. In this work, both the adsorption and diffusion behavior of CO_2_ to the polyaniline molecule were studied.

To estimate the effects of number of ionizable groups, and distribution of functional groups and adsorption sites on the sorption of H_2_O and CO_2_ on the polymers, the adsorption energy, charge transfer and band gap were calculated by using QM calculations based on DFT. The density functional was treated by the generalized gradient approximation (GGA) with exchange-correlation potential parameterized by Perdew-Burke-Ernzerhof (PBE) [43]. Geometry optimizations of polymer structures were performed with convergence tolerance of energy of 10^−5^ hartree and the maximal allowed force and displacement were 0.002 hartree/Å and 0.005 Å, respectively. A smearing of 0.005 hartree was applied to achieve accurate electronic convergence. The *k*-point was set to 12 × 1 × 1. Self-consistent field procedure was carried out with a convergence criterion of 10^−6^ a.u. A double numerical basis set plus polarization basis sets (DNP) were used. Gas physisorption on PANI was governed by the van der Waals (vdW) and electrostatic interaction. The adsorption energy *E*_ad_ was determined by:

Δ*E*_ad_ = *E*_polyaniline-analyte_ − (*E*_polyaniline_ + *E*_analyte_) (1)

where *E*_polyaniline-analyte_, *E*_polyaniline_, and *E*_analyte_ were, respectively, representing the total system potential energy of polyaniline-analyte system, isolated polyaniline system, and analyte molecule.

Based on the adsorption energy, charge transfer and band gap, the sensitivity of the polyaniline towards CO_2_ was evaluated, and the results were compared with the previous works. The sensitivity *S* was the key parameter for the design and evaluation of chemical sensing materials. In a humid environment,

CO_2_ + H_2_O ⇌ H^+^ + HCO_3_^−^ (2)

based on the Equation (2), the sensitivity of CO_2_ for the Na-SPANI/EB-PANI was predicted by the following equation:

(3) $$S_{\text{increasing}}=\frac{{\left(\Delta E_{ad}\times \Delta Q\times \Delta B\right)}_{\text{Na}-\text{SPANI}\_{\text{CO}}_{2}}\times {\left(\Delta E_{ad}\times \Delta Q\times \Delta B\right)}_{\text{Na}-\text{SPANI}\_H_{2}O}}{{\left(\Delta E_{ad}\times \Delta Q\times \Delta B\right)}_{\text{EB}-\text{PANI}\_{\text{CO}}_{2}}\times {\left(\Delta E_{ad}\times \Delta Q\times \Delta B\right)}_{\text{EB}-\text{PANI}\_H_{2}O}}$$

where ${\left(\Delta E_{\text{ad}}\times \Delta Q\times \Delta B\right)}_{\text{Na}-\text{SPANI}\_{\text{CO}}_{2}}$ and ${\left(\Delta E_{\text{ad}}\times \Delta Q\times \Delta B\right)}_{\text{EB}-\text{PANI}\_{\text{CO}}_{2}}$ were the sensitivity of Na-PSANI and EB-PANI towards CO_2_, respectively; ${\left(\Delta E_{\text{ad}}\times \Delta Q\times \Delta B\right)}_{\text{Na}-\text{SPANI}\_H_{2}O}$ and ${\left(\Delta E_{\text{ad}}\times \Delta Q\times \Delta B\right)}_{\text{EB}-\text{PANI}\_H_{2}O}\;$were the sensitivity of Na-PSANI towards H_2_O, respectively.

Because two adsorption sites were considered for the polyaniline, therefore, $\Delta E_{\text{ad}}\;$was the mean value of the adsorption energy of CO_2_ or H_2_O in EB-PANI or Na-SPANI. The Δ*E*_ad_ was calculated by:

(4) $$\Delta E_{\text{ad}}=\frac{E_{{\text{ad}}_{1}}+E_{{\text{ad}}_{2}}}{2}$$

where *E*_ad1_ was the adsorption energy of analyte adsorbed in site 1 of the polyaniline, and *E*_ad2_ was the adsorption energy of analyte adsorbed in site 2 of the polyaniline, according to Equation (1), the *E*_ad_ can be calculated.

Similarly, we defined the Δ*Q* as the following form:

(5) $$\Delta Q=\frac{\left|Q_{1}\right|+\left|Q_{2}\right|}{2}$$

|*Q*_1_| was the absolute value of the analyte adsorbed in site 1 and |*Q*_2_| was the absolute value of the analyte adsorbed in site 2.

The Δ*B*, which can be estimated from the band gap of isolated polyaniline,

(6) $$\Delta B=\frac{\left|B_{1}-B_{0}\right|+\left|B_{2}-B_{0}\right|}{2}$$

*B*_0_ was the band gap of the isolated polyaniline system, and *B*_1_ was the band gap of analyte adsorbed in site 1 of the polyaniline, the same as to *B*_2_.

Except for sensitivity, adsorbability was another important performance that would influence the sensing properties and the working range of sensing materials. The adsorbability of H_2_O and CO_2_ onto polymer in macroscopic scale was studied with MM. The polymer chains were initially minimized by MM and then by canonical ensemble (NVT) for 10 ps at 298 K. An amorphous unit cell containing 20 monomers was constructed by “Amorphous Cell” module with a low initial density of 0.20 g/cm^3^ [39]. The polymer was packed in periodic boundary condition (PBC) to reduce the effect of the surface [40]. Combining the advantage of our previous method [39] and Ostwal *et al.*’s [12] method, a new equilibrating protocol was produced in this study. Firstly, the energy of the amorphous polymer box was minimized by MM. This was followed by compressing at high pressures 1 GPa, 0.5 GPa and 0.0001 GPa using isothermal–isobaric ensemble (NPT) ensemble for 20 ps, 50 ps and 200 ps, respectively, to increase the polymer’s density (*ρ*) as close as possible to their experimental value at 298 K. The equilibration was attained by running a stepwise procedure of NVT of heating from 298 K to 698 K and then gradually cooling down to 298 K in a gradient of 50 K; each step was 50 ps and five cycles of the annealing dynamics were performed. The annealing dynamics was followed by an NPT run at 1 atm and 298 K, and in duration of 1000 ps. The total simulation time for the equilibration process was 5.27 ns. The simulation time for the equilibration process we took in the simulations was far larger than the 1.43 ns carried out by Ostwal *et al.* [12]. Subsequently, the output of the dynamics was used to calculate the adsorption isotherm of polymer system. According to our previous work [18], the Langmuir adsorption isotherm can be used for describing the adsorption process:

(7) $$A\;+\;<S^{*}>\begin{array}{c} k_{f}\\ ⇌\\ k_{b} \end{array}<SA>$$

where *A* was the analyte particles; <S^*^> was uniform distribution of immobile reaction sites; <*SA*> was the filled analyte (H_2_O or CO_2_) particle sites; and *k*_f_ and *k*_b_, were the forward and backward reaction rates, respectively.

Based on the self-diffusivity of H_2_O in EB-PANI and Na-SPANI (III) calculated by our previous work [44], the transport of CO_2_ in EB-PANI and Na-SPANI (III) affected by the ionizable groups were studied here. Ten CO_2_ molecules were added to the simulation box containing 80 monomers. The polymer models were then compressed and equilibrated/relaxed using the same methodology as addressed for adsorption isotherm. Then, NVT dynamics at 298 K was run for 7 ns, after the dynamics the system was used to calculate the diffusion coefficient (*D*). Comparing with 30 monomers Ostwal *et al.* [12] used to estimate the self-diffusivity of water in PANI-Cl and PANI-Br, 80 monomers of our protocol was large enough. All MM/MD simulations for adsorption isotherm and diffusion coefficient were run with a 1.0 fs time step. The temperature and pressure were controlled by the Berendsen’s method using a half-life for decay to the target temperature of 0.1 ps (decay constant) and 0.1 ps for the pressure scaling constant. The non-bonded electrostatic and van der Waals forces were controlled by “Ewald” at “Fine” quality and “Atom based” with a cutoff value of 10.5 Å for sorption calculations and 15.5 Å for diffusion calculations, respectively. More detailed settings for geometry optimization and diffusion coefficient calculation were listed in Table 1. The *D* was then calculated from the mean-square displacement (MSD) of the CO_2_ molecules using [13],

(8) $$D=\underset{t\to \infty }{\lim}\frac{1}{6t}\langle \left|R\left(t\right)-R\left(0\right)\right|{}^{2}\rangle$$

where *D* was diffusion coefficient, 〈•〉 represented an average over all the CO_2_ molecules, and *R*(*t*) was the position vector of a molecule at time *t*.

## 3. Results and Discussion

The adsorption energy (Δ*E_ad_*), charge transfer and band gap of H_2_O and CO_2_ molecules in different polyaniline systems, which can be used to evaluate the effect of the different amount/distribution of the functional group and the adsorption position on the adsorption mechanism, are summarized in Table 2. Comparative analysis of the Δ*E_ad_* for EB-PANI CO_2__1 (−0.054 eV), Na-SPANI (I) CO_2__1 (−0.115 eV) and Na-SPANI (III) CO_2__1 (−0.189 eV) implies that the Δ*E_ad_* is proportional to the amount of –SO_3_^−^Na^+^. In addition, the Δ*E_ad_* of Na-SPANI (I) CO_2__2 is −0.111 (eV), yet it is −0.32 (eV) for Na-SPANI (II) CO_2__2, from which we can deduce that the Δ*E_ad_* is also dominated by the distribution of functional group on the phenyl ring. Furthermore, Δ*E_ad_* of site 1 to site 2 shifts from −0.054 (eV) to −0.082 (eV) for EB-PANI and from −0.189 (eV) to −0.564 (eV) for Na-SPANI (III), indicating that the adsorption position is another factor attributing to the difference of the Δ*E_ad_*.

Similar to the adsorption of CO_2_, the Δ*E_ad_* of H_2_O adsorbed in these four types of polyaniline are also influenced by the factors discussed above. For instance, the Δ*E_ad_* of EB-PANI H_2_O_1, Na-SPANI (I) H_2_O_1, Na-SPANI (II) H_2_O_1 and Na-SPANI (III) H_2_O_1 are −0.435 (eV), −0.501 (eV), −0.491 (eV), and −1.06 (eV), respectively. Either the increased number or the position change of —SO_3_^−^Na^+^ on the phenyl ring will lead to the variation of Δ*E_ad_*. Compared with site 1, the Δ*E_ad_* for EB-PANI H_2_O_2, Na-SPANI (I) H_2_O_2, Na-SPANI (II) H_2_O_2 and Na-SPANI (III) H_2_O_2 are −0.463 (eV), −0.587 (eV), −0.899 (eV) and −1.188 (eV) respectively, supporting the effect that the adsorption sites have on the Δ*E_ad_*. Comparative results of Δ*E_ad_* for CO_2_ and H_2_O adsorption conclude that the polymer chains are more sensitive to H_2_O. We believe that it is the hydrogen bond formed by the interplay between H_2_O and the unsaturated nitrogen atoms that leads to the more pronounced interaction. In addition, Na-SPANI (III) has superior adsorption capacity with both CO_2_ and H_2_O over EB-PANI, Na-SPANI (I) and Na-SPANI (II). Moreover, site 2 exhibits better adsorption performance than site 1.

Mulliken charge analyses of interaction between analytes and PANI complexes are also given in Table 2. The interaction is established when the electrons transfer from analyte molecules to PANI, charge transfer from analyte to PANI is positive while from PANI to analyte is negative. In the case of CO_2_ adsorbed at these two different sites, the charge transfer from polymer to the analytes is small, and the change of band gap is negligible in the EB-PANI system. After introducing the –SO_3_^−^Na^+^, the charge transfers of these three Na-SPANI systems become relatively stronger than that of EB-PANI, and it is the strongest for Na-SPANI (II) CO_2__2 (0.013 |e|) and Na-SPANI (III) CO_2__2 (0.014 |e|). All of these results reveal that the adsorption site is one of the key factors affecting the charge transfer. As for H_2_O, almost all the charges transfer from polymer to H_2_O, which is similar to the situation of polymer-CO_2_ complex, but the degree of charge transfer is much larger since the minimum value is −0.011 e, except for Na-SPANI (II) H_2_O_2 (0.021 |e|) and Na-SPANI (III) H_2_O_2 (0.014 |e|). It is clearly proved that the type of gas molecules is another vital factor affecting the charge transfer. Notably, dramatic changes of band gap will take place when CO_2_ or H_2_O interacts with Na-SPANI (II) or Na-SPANI (III). Generally, for H_2_O and CO_2_ adsorbed at site 2 close to the –SO_3_^−^Na^+^, both of charge transfer are positive, for the reason that –SO_3_^−^Na^+^ is a strong electron-withdrawing group. Both of the adsorption energies and charge transfer show that site 2 of Na-SPANI (III) is most sensitive towards the analytes.

The increasing sensitivity of Na-SPANI (I), Na-SPANI (II) and Na-SPANI (III) to EB-PANI are summarized in Table 3. The sensitivity of Na-SPANI/EB-PANI towards CO_2_ are improved about two orders of magnitude, the Na-SPANI (III)/EB-PANI in particular reach the value of 355. According to the sensing mechanism and Equation (2), the results indicate that Na-SPANI can detected much lower concentration of CO_2_ than EB-PANI. That is, Na-SPANI can work in a higher pH working range, the work range can change from pH 2.0–4.0 for EB-PANI to pH 4.0–6.0 for Na-SPANI. This shift is consistent with the experimental observation reported by Doan *et al.* [33] Thus, our methods for evaluating the sensitivity of polyaniline to CO_2_ are realistic and accurate.

**Figure 4:**
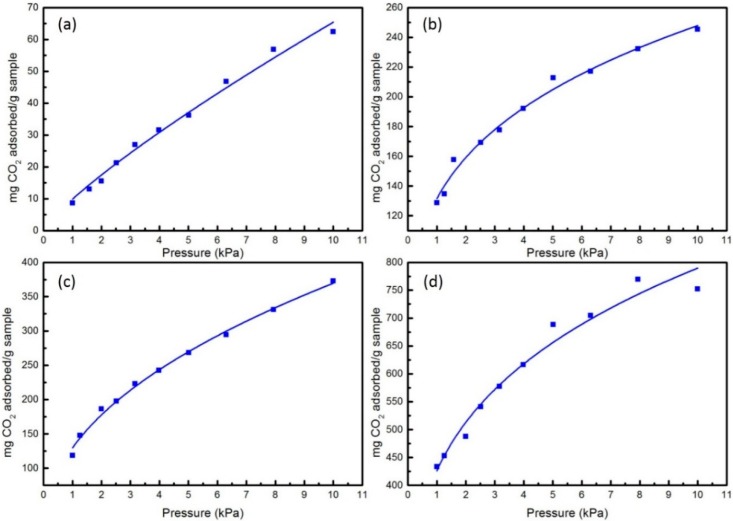
The computed sorption isotherms of CO_2_ in (**a**) EB-PANI; (**b**) Na-SPANI (I); (**c**) Na-SPANI (II); (**d**) Na-SPANI (III) at 298 K.

**Figure 5:**
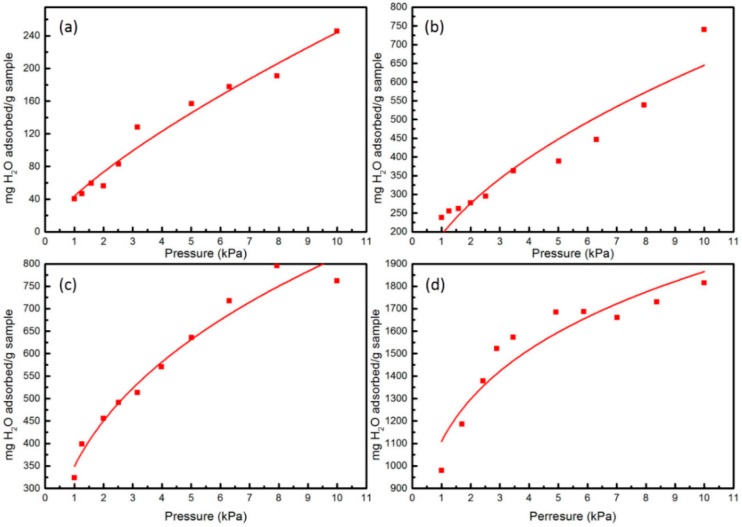
The computed sorption isotherms of H_2_O in (**a**) EB-PANI; (**b**) Na-SPANI (I); (**c**) Na-SPANI (II); (**d**) Na-SPANI (III) at 298 K.

Except the sensitivity, the knowledge of adsorption quantity of analyte molecules in a polyaniline is an important aspect for polyaniline as a sensor for the reason that it can directly affect the formation of H_2_CO_3_. According to Equation (7), when the adsorption reaches to equilibrium, the adsorption rate and desorption rate will be equal, and a Langmuir type adsorption isotherm can be obtained for these adsorbents. Figure 4 and Figure 5 give the sorption isotherms of CO_2_ and H_2_O in these four types of PANI at 298 K, respectively. Obviously, from Figure 4 and Figure 5, we can see that with the pressure increased from 1 kPa to 10 kPa, the adsorption quantity of these four kinds of adsorbent increase in turn, no matter for CO_2_ or H_2_O. Simultaneously, the sorption capacities of the systems tend to be saturated when the pressure increased to a greater extent. For the sorption of CO_2_ shown in Figure 4, there is a significant difference among the sorption isotherms in EB-PANI, Na-SPANI (I), Na-SPANI (II) and Na-SPANI (III). During the given pressure from 1 kPa to 10 kPa, the Na-SPANI (II) and Na-SPANI (III) at 1 kPa are able to adsorb 118 and 433 mg/g, while for EB-PANI and Na-SPANI (I), they are 8.6 and 128 mg/g, respectively. Obviously, the Na-SPANI (II) and Na-SPANI (III) have a much higher adsorption quantity than EB-PANI and Na-SPANI (I). Similarly, the adsorption quantity of Na-SPANI (III) for H_2_O at a pressure of 1 kPa is about 979 mg/g (Figure 5). For EB-PANI, Na-SPANI (I) and Na-SPANI (II), the amounts are 40, 237, and 323 mg/g, respectively. The adsorption quantity of 40 mg/g for EB-PANI is in accordance with the reported result of 42 mg/g [12,45]. It is clear to see that the adsorption quantities of these four adsorbents for H_2_O are much higher than that of CO_2_. These results are in agreement with the above quantum computation, in which H_2_O interacts stronger with polyaniline than CO_2_. Furthermore, Na-SPANI (III) has the strongest adsorption energies towards the analytes, and this is further supported by the following diffusion coefficient calculation.

The diffusion coefficients of H_2_O in the EB-PANI and Na-SPANI (III) are estimated, and the values are *D*_EB-PANI_ = 6.95 × 10^−9^ cm^2^/s and *D*_Na-SPANI(III)_ = 6.15 × 10^−9^ cm^2^/s respectively [44], which are close to experimental data *D*_PANI–Cl_ = 3.14 × 10^−9^ cm^2^/s and *D*_PANI–Br_ = 2.43 × 10^−9^ cm^2^/s that were calculated by Ostwal *et al.* [24] for diffusion of water vapor in doped PANI. Comparing with the estimated diffusion coefficients of water in the two doped polymers are then *D*_PANI–Cl_ = 5.1 × 10^−8^ cm^2^/s and *D*_PANI–Br_ = 4.16 × 10^−8^ cm^2^/s [12], which are larger than the experimental data by about one order of magnitude. Our results are more consistent with the experiment data, which could be explained by the following two reasons: our atomistic models are much larger and simulation time for equilibration process is longer. The size of the model is a prime factor in determining diffusion coefficients of small gas molecules in the polymer [46].

**Figure 6:**
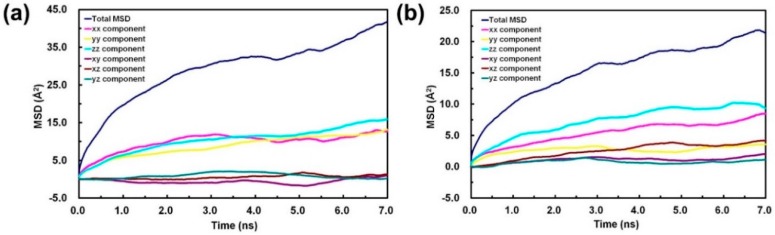
Mean-square displacement (MSD) of CO_2_ in (**a**) EB-PANI and (**b**) Na-SPANI (III) as a function of simulation time.

**Figure 7:**
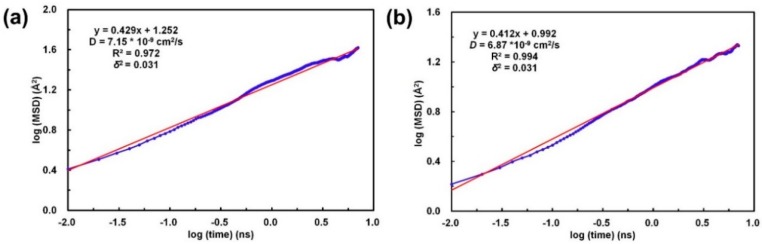
The relationship between log (MSD) and log (t) of CO_2_ diffusion in (**a**) EB-PANI and (**b**) Na-SPANI (III).

Based on the calculation results of the H_2_O diffusion in EB-PANI and Na-SPANI (III), the diffusivities of CO_2_ in EB-PANI and Na-SPANI (III) are calculated in this study. Figure 6 shows the relationships between the MSD and time of CO_2_ in each directions. The computation of the logarithmic MSD and simulation time is performed to calculate the diffusion coefficients. According to Equation (8), the relationship between log (MSD) and log (t) of CO_2_ diffusion in EB-PANI and Na-SPANI (III) presented in Figure 7 should be a linear function. From Figure 7, we can see that the log (MSD) *versus* log(t) is approximately a linear relationship, which means the diffusive transport is up to equilibrium. By fitting the curves, the slope *a*, the correlation coefficient *R*^2^_EB-PANI_ = 0.972 and *R*^2^_Na-SPANI_ = 0.994, and the variances of both system *δ*^2^ = 0.031 are obtained. The differential approximation in the Equation (8) is substituted by the ratio of log (MSD) *versus* log (t), namely, the slope *a*. Then, Equation (8) can be simplified to *D* = *a*/6; therefore, the diffusivities of CO_2_ calculated here are *D*_EB-PANI_ = 7.15 × 10^−9^ cm^2^/s (Figure 7a) and *D*_Na-SPANI_ = 6.87 × 10^−9^ cm^2^/s (Figure 7b). The ratio of CO_2_/H_2_O in EB-PANI system is 7.15 × 10^−9^/6.95 × 10^−9^ = 1.03, while in the Na-SPANI (III) system, it is 6.87 × 10^−9^/6.15 × 10^−9^ = 1.12. Diffusion coefficient of CO_2_ is higher than that of H_2_O due to the nonpolar nature of the CO_2_ structure, which makes it easier to diffuse. Meanwhile, the hydrogen bond formed by the interaction of H_2_O with polyaniline is stronger than the dipole formed by the interaction of CO_2_ with polyaniline. Moreover, the ratio of diffusion coefficient of CO_2_/H_2_O in Na-SPANI (III) is larger than the one in EB-PANI. It is the high-free-volume of EB-PANI that makes the system denser after introducing –SO_3_^−^Na^+^ groups, therefore limiting the diffusion of H_2_O molecules. On the basis of the diffusion coefficients calculation, it is concluded that Na-SPANI (III) polymers can adsorb more H_2_O and CO_2_ molecules into the polymer matrix than the EB-PANI due to the more compact Na-SPANI (III) system.

## 4. Conclusions

By using a combination of QM and MD techniques, we have investigated how the ionizable group (–SO_3_^−^Na^+^) affects the material properties of polyaniline for gas sensing applications. The simulation results show that two effects (position and amount) are associated with the sulfate substituent. Through adsorption of CO_2_ and H_2_O in polyaniline, we observe that the adsorption energy, Mulliken charge and band gap of the analytes could affect the sensitivity of the polyaniline. In addition, the sorption isotherm of the analytes in the polyaniline are also studied. All of the adsorption energy, Mulliken charge and sorption isotherm of the analytes are relatively sensitive to both position and the amounts of –SO_3_^−^Na^+^. By contrast, the band gap of polyaniline exhibits small changes responding to the different molecular structures. We also study the changes in their properties with changes in the adsorption site of the analytes on the polymer chain. It is evident that the adsorption energy is significantly affected by the position of the adsorption site when in the presence of –SO_3_^−^Na^+^, and the sensitivity of the polyaniline can be improved by introducing –SO_3_^−^Na^+^. We also develop the molecular models, which are capable of estimating the self-diffusivity of the gas molecules in the polymer matrix. The data of the diffusion coefficient demonstrates that the –SO_3_^−^Na^+^ groups can notably affect the gas transport properties of polyaniline. Comparative studies indicate that the effect of ionizable groups on polyaniline as gas sensing materials for the polar gas molecule (H_2_O) is more significant than that for the nonpolar gas molecule (CO_2_). These results indicate that Na-SPANI (III) is a good candidate for developing a CO_2_ sensor. This conclusion agrees very well with the result of previous experimental studies. Our findings provide important information to reveal the underlying action mechanisms of ionizable groups on the sensing properties of polyaniline.

## Figures and Tables

**Table 1:** Settings for geometry optimization and equilibration process of polymer systems and for the MD simulation of CO_2_ diffusion in polymer systems.

Geometry Optimization	Equilibration Process of Structure		
Forefield: COMPASS	Step	Simulation conditions	Time (ps)
Quality: Fine			
Summation method: Ewald for electrostatic and atom base for van der Waals (vdW)	1	NPT, 1 GPa, 298 K	20
Cutoff distance: 10.5 Å for sorption and 15.5 Å for diffusion	2	NPT, 0.5 GPa, 298 K	50
Algorithm: smart	3	NPT, 0.0001 GPa, 298 K	200
‘Fine’ convergence tolerance	4	A stepwise procedure of NVT of heating from 298 K to 698 K and cooling from 698 K down to 298 K by a step of 50 K	50 ps/stepwise
			5 cycles
Energy (kcal/mol): 1 × 10^−4^	5	NPT, 1 atm, 298 K	1000
Buffer width: 0.5 Å	The total simulation time for the equilibration process is 5.27 ns		
Spline width: 1 Å	MD simulation for CO_2_ diffusion in the polymer system		
Displacement (Å): 5 × 10^−5^		NVT, 298 K	7000
Max. iterations: 50,000			

**Table 2:** Adsorption energy (Δ*E_ad_*), charge transfer (*Q*) and band gap (*B*) of H_2_O and CO_2_ molecules on polymers.

Species	Δ*E_ad_* (eV)	*Q* (|e|)	*B* (eV)
EB-PANI			1.44
Na-SPANI (I)			1.44
Na-SPANI (II)			1.30
Na-SPANI (III)			1.29
EB-PANI CO_2__1	−0.054	−0.001	1.43
EB-PANI CO_2__2	−0.082	−0.001	1.45
Na-SPANI (I) CO_2__1	−0.115	−0.006	1.42
Na-SPANI (I) CO_2__2	−0.111	−0.005	1.42
Na-SPANI (II) CO_2__1	−0.112	−0.005	1.33
Na-SPANI (II) CO_2__2	−0.32	0.013	1.33
Na-SPANI (III) CO_2__1	−0.189	−0.006	1.34
Na-SPANI (III) CO_2__2	−0.564	0.014	1.34
EB-PANI H_2_O_1	−0.435	−0.017	1.46
EB-PANI H_2_O_2	−0.463	−0.014	1.42
Na-SPANI (I) H_2_O_1	−0.501	−0.015	1.42
Na-SPANI (I) H_2_O_2	−0.587	−0.014	1.42
Na-SPANI (II) H_2_O_1	−0.491	−0.011	1.33
Na-SPANI (II) H_2_O_2	−0.899	0.021	1.32
Na-SPANI (III) H_2_O_1	−1.06	−0.013	1.24
Na-SPANI (III) H_2_O_2	−1.188	0.014	1.36

**Table 3:** The evaluated increasing sensitivity of three types of Na-SPANI/EB-PANI towards CO_2_.

Polymers	S_increasing_
Na-SPANI (I)/EB-PANI	41.4
Na-SPANI (II)/EB-PANI	171.3
Na-SPANI (III)/EB-PANI	355.7
